# Investigating persistent measles dynamics associated with population immunity in Chad: a national secondary data model analysis from 2011 to 2025

**DOI:** 10.1016/j.ijregi.2025.100684

**Published:** 2025-06-15

**Authors:** Gilson Kipese Paluku, Balcha Girma Masresha, Antoinette Demian Mbaïlamen, Tamadji Mbaihol, Djalal Mohamed El-hafiz, Thierry Vincent, Jacques L. Tamuzi, Patrick DMC Katoto, Charles S. Wiysonge, Blanche-Philomene Melanga Anya

**Affiliations:** 1 Team leader of Universal Health Coverage/Communicable and Non-communicable diseases (UCN) at the WHO Office, N’djamena, Chad; 2 Coordinator of measles and rubella elimination in the WHO Africa Region, Brazzaville, Congo; 3 Director of the Expanded Program on Immunization (EPI) and epidemiological surveillance, N’djamena, Chad; 4 WHO Country Office, N’djamena, Republic of Chad; 5 Gavi, N’djamena, Chad; 6 Division of Epidemiology and Biostatistics, Department of Global Health, Faculty of Medicine and Health Sciences, Stellenbosch University, Cape Town, South Africa; 7 Centre for Tropical Diseases and Global Health, Department of Medicine, Catholic University of Bukavu, Bukavu, Democratic Republic of the Congo; 8 Cochrane South Africa, South African Medical Research Council, Cape Town, South Africa; 9 World Health Organization Regional Office for Africa, Brazzaville, Congo

**Keywords:** Measles, Population immunity, Recurrent outbreaks, Chad

## Abstract

**Objectives:**

To model the population measles immunity gaps in a birth cohort of children aged 0-14 years in Chad from 2011 to 2025.

**Methods:**

This study used the Centers for Disease Control and Prevention–established population modeling analysis measles immunity profile approach, including data published by World Health Organization, United Nations Children’s Fund, and United Nations Population. Excel and R Software were used to build a stacked bar chart of population immunity using immunity by measles-containing vaccine (MCV) dose 1 (MCV1) (%), by MCV dose 2 (MCV2) (%), by supplemental immunization activities (%), by maternal antibodies (%), and the rate of unprotected individuals against measles.

**Results:**

The population immunity gaps ranged from 5% to 6% in the cohort of children born in 2011-2013. We estimated the immunity gaps to be 13% in 2014-2015. Our results revealed an increase in population immunity gaps of 21% in 2016 and 2017 and 20% computed in 2018. From 2019 to 2022, the immunity gaps are ranged from 6% to 8%, correlating with a decrease of three to four times less than 2016-2018. A significant increase in immunity was observed in 2023, 2024, and 2025, with 46%, 53%, and 36%, respectively. The overall cohort showed a significative cumulative number of cases of measles susceptibility correlated with population immunity gaps. The findings also revealed insufficient MCV1 coverage, a late introduction of MCV2, poor MCV2 coverage, and a high MCV1/MCV2 abandonment rate over time.

**Conclusions:**

The risk of recurrent measles outbreaks remains high because of high population immunity profile gaps, increasing the population susceptible. To address immunity gaps, it is necessary to increase coverage by routine vaccination and high-quality supplemental immunization activities in Chad.

## Highlights

• Measles outbreaks are recurrent in Chad.
• Nearly 33% children in Chad are underimmunized.
• Significative cumulative cases of measles susceptibility in Chad.
• High population measles immunity profile gaps in Chad.

## Introduction

An estimated 107,500 measles fatalities occurred worldwide, predominantly among unvaccinated or under-vaccinated children under the age of 5 years [1]. The highest incidence is in the World Health Organization (WHO) African region, with measles incidence increasing from 69.2 per 1 million population in 2017 to 81.9 in 2021 [2]. The number of estimated annual measles cases and deaths increased 22% and 8%, respectively. However, measles vaccination averted more than 60 million deaths between 2000 and 2023 [1]. The growing number and size of measles outbreaks is concerning because it represents a future public health burden in measles cases and fatalities, secondary sequelae, and greater health care utilization and costs, which the current public health system and funding might not be able to manage [3].

Measles outbreaks are recurrent in Chad, where they are one of the main causes of death among children. From January 1, 2024 to May 5, 2024, Chad reported 5631 probable measles cases, leading to 20 fatalities [4]. Chad has been experiencing recurring measles epidemics for several years. Recent statistics indicated a fluctuation of epidemics throughout 16-78 districts annually in Chad. With the increasing complexity of humanitarian access in the Sahel, nearly 33% of children in Chad are underimmunized [5]. Chad’s vaccination coverage rates are suboptimal, and the country is a long way from meeting the 90% target coverage rates for vaccination in children [4]. Although recent data showed that vaccination against measles increased by 8% in Chad [6], this is still low compared with the gap in vaccination coverage. Furthermore, the ongoing influx of refugees, the majority of whom are zero-dose and hence vulnerable; some arriving after the supplemental immunization activities (SIAs) may lower the population immunity. Studies have also shown that multiple factors could influence recurrent measles outbreaks, including suboptimal SIAs, limited access to health care services, contact with infected individuals, and shorter duration of maternal immunity [7,8]. To achieve herd immunity for measles, the required vaccination coverage against measles ranges from 93% to 95%, with two doses of measles vaccine to prevent outbreaks [1,9,10]. However, only 81% of 2-year-old children worldwide receive the first dose and 71% the second dose, resulting in major gaps in coverage [11,12].

In fact, people who contract measles are known to be susceptible to the disease, which means they are either unvaccinated or vaccinated but not immune. People who are not immune to measles could be directly measured using serosurveys. However, serosurveys need a significant amount of time and resources and cannot be undertaken on a regular basis. In comparison, measles immunity profiles use widely available vaccination coverage data to predict immunity gaps [13]. Measles immunity profiles calculate and display the proportion of people protected by vaccination by birth cohort, based on coverage of measles-containing vaccine (MCV) dose 1 (MCV1), MCV dose 2 (MCV2), and SIAs. Compared with serosurveys, the immunity profile is a faster and less expensive tool to measure population immunity gaps, and vaccination programs can use it to estimate susceptibilities. Percentages of children immunized with MCV1, MCV2, and SIAs against measles, as well as the percentage of those susceptible to measles, categorized by year of birth were calculated using the measles immunity profile methodology established by the Centers for Disease Control and Prevention (CDC) [13,14]. In countries where routine immunization cannot maintain high levels of immunity to measles, the WHO recommends monitoring the risk of measles outbreaks by estimating the accumulated number of measles-susceptible preschool-aged children and conducting SIAs before that number reaches the size of one birth cohort [15]. This is applicable in Chad, where several measles outbreaks are recurring over time, as shown in the study justification. However, no published study has analyzed the recent trends of population measles immunity levels that may influence recurrent measles outbreaks in Chad. This study uses a Chadian birth cohort to depict the population measles immunity gaps in children aged 0-14 years from 2011 to 2025.

## Study justifications

**Figure 1:**
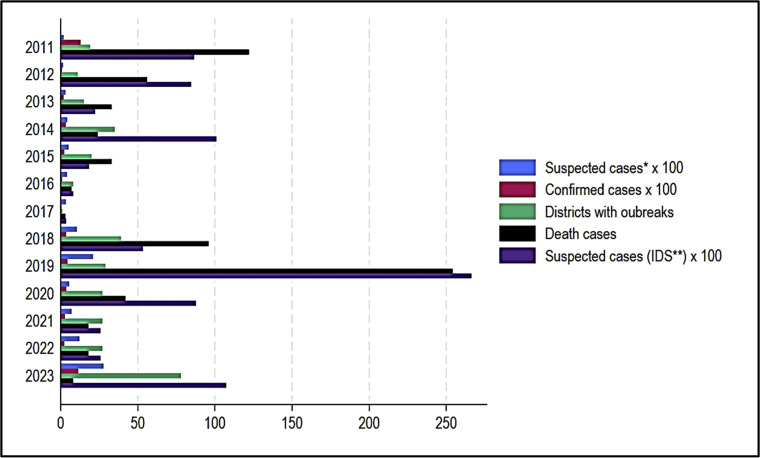
Suspected and confirmed cases of measles, epidemic districts, and deaths in Chad between 2018 and 2023.

Note: *Data from case-based surveillance database; ** Data from the IDS database.

Chad has been experiencing outbreaks of measles on a regular basis for several years. For example, Figure 1 displays the number of measles cases and the districts that were affected by the epidemic from 2011 to 2023. From 2011 to 2023, Chad has recorded ranges of 153 to 2766 suspected measles cases (data from case-based surveillance database) and 349 to 10,732 (data from Integrated Data Surveillance (IDS) database), nine to 1130 confirmed measles cases, one to 78 districts affected with measles outbreaks, and three to 254 deaths associated with measles (Figure 1). It is known that people who get measles are those susceptible to measles [1], i.e. unvaccinated or vaccinated but not immune [1]. People who are not immune to measles could be measured directly by serosurveys. Nonetheless, serosurveys are laborious and resource-demanding, making frequent implementation impractical. In contrast, measles immunity profiles assess immunity gaps based on accessible vaccination coverage data. Measles immunity profiles assess and indicate the proportion of individuals safeguarded by vaccination according to birth cohort, based on the coverage of the first and second doses of MCV and SIAs [14]. This approach offers a more efficient and cost-effective method of evaluating immunity gaps than serosurveys, and it can be used by vaccination programs to estimate susceptibilities. This methodology will be implemented to ascertain the quantity of susceptible individuals in this article.

## Methods

### Study design

This was a population modeling analysis using vaccination coverage secondary data published jointly by the WHO and the United Nations Children’s Fund (UNICEF) and United Nations (UN) population in Chad [[16], [17], [18]].

### Study population

This study included a birth cohort of children aged 0-14 years according to the UN population [18].

### Study setting

The Republic of Chad is a landlocked country in central Africa. It is bordered by Cameroon, the Central African Republic, Libya, Niger, Nigeria, and Sudan. It is a low-income country with a gross domestic product per capita of US$ 686 (2021) and a population of 17.2 million (2021) [4]. The population is unevenly distributed across the country, with half residing in the southern 20% and the remainder dispersed throughout the remaining 80% of the country. A high proportion (80%) lives more than 5 km from a health facility in Chad. International partners have played a crucial role in Chad’s efforts to reach zero-dose children by supporting the Ministry of Public Health and Prevention.

Global geospatial data from 2019 identified Chad as having one of the highest proportions of zero-dose children aged under 12 months. Children in the large remote rural areas of northern Chad, rural non-remote areas, and conflict-affected areas are particularly affected, including those in nomadic communities [19]. Urban zones also have a sizable number of zero-dose children [20]. Estimates for 2022 put the number of zero-dose children at 188,500, with an additional estimate of 122,935 under-vaccinated [20].

### Data collection and management

We collected secondary population data from the WHO and UNICEF vaccination coverage estimates called WHO/UNICEF joint estimates of national immunization coverage (WUENIC) from 2011 to 2023, UN population, and those of supplementary immunization activities conducted during the same period, and routine immunization [16,18]. Data collected included dates of surveys, number of children born, target and affected population for measles immunization, MCV doses 1 and 2, SIAs rate, maternal antibody (Ab) rate, no protection against measles rate, confirmed and suspected measles cases, measles incidence, and number of children without SIAs. An Excel spreadsheet was used to collect data and import it to Stata 18 MP for codification and cleaning.

### Assumptions and analysis

The probability that a child will be reached by successive immunization services, given their vaccination history, is addressed by the correlation between doses. WHO recommends that a dose be recorded as MCV2 for routine immunization only if the child has received the first documented dose of MCV1. Consequently, the MCV2 is contingent upon the MCV1 [21]. The likelihood of being vaccinated is not influenced by the child’s vaccination status before the SIA if each child has an equal chance of receiving the vaccine at each SIA. This finding is in stark contrast to the prevalent observation that children who were vaccinated by earlier health services are frequently the first to be reached by later SIAs. We presupposed that each vaccination opportunity initially results in the revaccination of children who have already been vaccinated. Vaccination of unvaccinated children only occurs when coverage exceeds previous opportunities. This assumption was made to more accurately represent the dependency between vaccination opportunities [14]. The “dependent scenario” and the “independent scenario” denote the boundaries of a range within which the actual correlation between the probability of receiving a subsequent dose and one’s previous vaccination status is determined [14]. We used the “dependent scenario” to offer a prudent assessment of the vaccination program’s impact and to mitigate the risk of overestimating immunity and postponing response activities because administrative vaccination coverage is frequently overestimated [14].

We also computed the additional percentage of children vaccinated by sub-national SIAs based on SIA coverage and the proportion reached during earlier vaccination chances instead of averaging coverage at the national level and “diluting” the impact in the sub-national area. For phased SIAs, we estimated individually the percentage of extra children vaccinated in the areas targeted by each phase and aggregated the impact at the national level. This is to illustrate that the percentages of people immunized with MCV1, MCV2, and SIAs against measles and the percentage of people vulnerable to measles by year of birth using the measles immunity profile method established by the CDC [13,14]. The immunity profile was created at any time using the CDC model, as long as the input data were accessible. When built for a future date, the profile reflects immunity gaps by cohort before a planned SIA or an increase in population immunity after a planned SIA.

When data from the vaccination coverage survey (VCS) of the SIAs became available, they supplanted administrative data [14]. If the VCS was not completed or conducted without available data, then administrative VCS data were used. In this instance, administrative data were restricted to 95%, unless subsequently the VCS demonstrated coverage beyond 95% [14]. Formulas based on the assumption that previously vaccinated children are first reached by a subsequent vaccine dose:

%ofpersonsimmunefromMCV1=MCV1×MCV1VE

$$\begin{array}{ccc} & & \%\;of\;additional\;persons\;immune\;from\;MCV2\\ & & =\left\{ \begin{array}{c} \left(MCV1-MCV1\times MCV1\;VE\right)\times \frac{MCV2}{MCV1}\times MCV2\;VE,\;when\;MCV2<MCV1,\;\\ \;\\ \left(MCV1-MCV1\times MCV1\;VE\right)\times MCV2\;VE+\left(MCV2-MCV1\right)\times MCV2\;VE,\;when\;MCV2\geq MCV1 \end{array}\right.\left[14\right] \end{array}$$

$$\begin{array}{ccc} & & Cumulative\;percentage\;vaccinated\;after\;SIA_{n}\\ & & =\left\{ \begin{array}{c} cumulative\;percentage\;vaccinated\;before\;SIAn,when\;SIA_{n}<cumulative\;\%\;vaccinated\;before\;SIA_{n}\;\\ \;\\ SIA_{n}\;coverage\times \%\;population\;targeted+cumulative\;precentage\;vaccinated\;before\;SIA_{n}\times \left(1-\%\;population\;targeted\right)\;\left[14\right]\;\\ \;\\ when\;SIA_{n}\;coverage\geq cumulative\;\%\;vaccinated\;before\;SIA_{n} \end{array}\right. \end{array}$$

$$\begin{array}{ccc} & & \%\;of\;additional\;persons\;immune\;from\;SIA_{n}\\ & & =\left\{ \begin{array}{c} \left(cumulative\;\%\;vaccinated\;before\;SIA_{n}-cumulative\;\%\;immune\;before\;SIA_{n}\right)\times \frac{SIA_{n}\;coverage}{cumulative\;\%\;vaccinated\;before\;SIA_{n}}\times SIA\;VE\times \%\;population\;targted,\;\left[14\right]\;\\ \;\\ when\;SIA_{n}\;coverage<cumulative\;\%\;vaccinated\;before\;SIA_{n},\\ \;\\ \left(SIA_{n}\;coverage-cumulative\;\%\;immune\;before\;SIA_{n}\right)\times SIA\;VE\times \%\;population\;targeted,when\;SIA_{n}\geq cumulative\;\%\;vaccinated\;before\;SIA_{n}\; \end{array}\right. \end{array}$$

*MCV1: first routine dose of the MCV*
*MCV2: second routine dose of the MCV*
*SIA: supplementary immunization activities*
*VE: vaccine effectiveness*

R Software version 4.3.3. (shiny) was used to illustrate to build a stacked bar chart of population immunity using immunity by MCV1, by MCV2, by SIAs, by maternal Abs, and the rate of unprotected individuals against measles. The CDC Measles immunity profiles was used to plot these factors [13,14].

### Measles vaccine effectiveness

The effectiveness of the measles vaccine depends on the age of the person receiving it. A recent literature review found that the median vaccine effectiveness of a single dose of MCV is 84% when given to children aged 9 to 11 months and 92.5% when given to children aged 12 months and older. These results are based on trials that verified vaccination history and laboratory-confirmed cases [13,22]. Among children who do not develop immunity after the first dose of MCV, about 95% will develop immunity with a second dose. The vaccine is less effective (58%) when given before the age of 9 months [13,23]. Our assumptions about the effectiveness of MCV1 and MCV2 vaccines are based on the age at which the vaccine is administered according to the national immunization schedule [13]. SIAs typically cover a broad age range, from 9 months to 14 years, and in outbreak settings, as young as 6 months. The cohorts targeted by the SIAs were divided into age groups based on previous vaccination status and age at the time of the SIA, and the appropriate estimate of vaccine effectiveness was used [13].

### Ethical considerations

This study was reviewed and approved by the Chadian Ministry of Health and Public Health. Although secondary data were used, the study was conducted in accordance with the Helsinki Declaration and local institutional policies on human research. According to the Council for International Organizations of Medical Sciences, vulnerable individuals may include children in developing or resource-limited countries [24]. In its Declaration of Helsinki, the World Medical Association said that medical research with a vulnerable group is only permissible if it is appropriate to the group’s health needs or goals and cannot be conducted in a non-vulnerable group. Furthermore, this group should stand to gain from the information, practices, or actions that come from the study [25].

## Results

### Population characteristics

We use an estimated population of the measles-susceptible preschool-aged children in Chad as published by UN population, as shown in Table 1 [18].

**Table 1:** Estimated number of persons by year of age (i.e. <1 year, 1 year to <2 years, etc.) published in World Population Prospects, 2019 Revision by United Nations Population Division.

Age in years	Total number of persons in the age group
0	761,163.5
1	731,342.5
2	710,443.0
3	686,804.5
4	662,774.5
5	641,310.0
6	617,581.0
7	594,824.5
8	576,526.0
9	562,420.0
10	548,582.5
11	531,287.0
12	514,180.0
13	497,071.5
14	479,495.5

### Characteristics of included surveys

Children in all Chad provinces between the ages of 6 months and 9 years were the target demographic for the measles WUENIC, which ran from 2011 to 2025 (Table 1).

### Population immunity gaps and measles vaccination coverage

**Figure 2:**
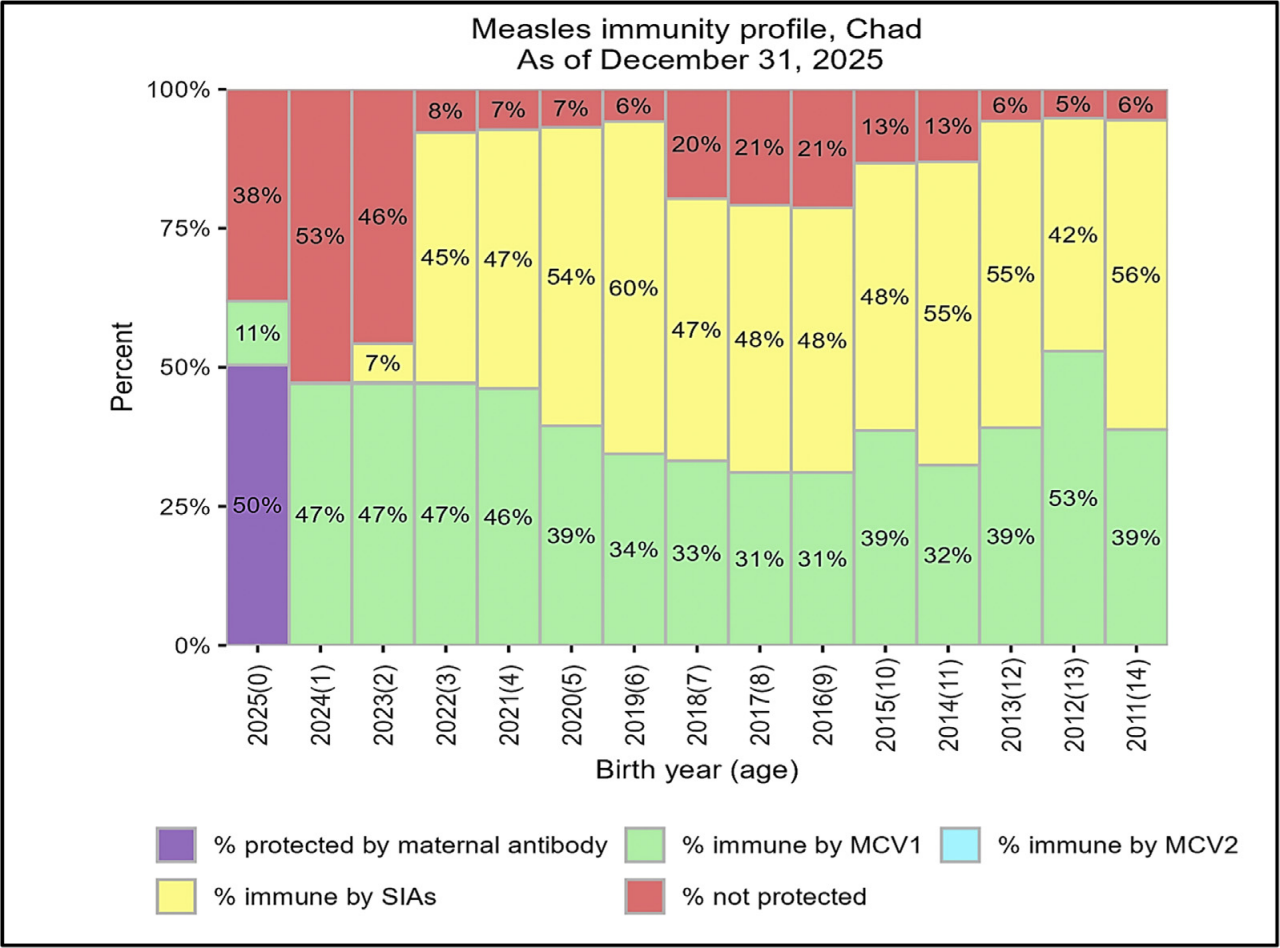
Measles population immunity profile from 2011 to 2025 showing estimated measles immunity gaps in red horizontal bars for each birth cohort in Chad.

MCV, measles-containing vaccine; SIAs, supplementary immunization activities.

Table 2 describes the cohort for birth children, including immunity by MVC1, by MVC2, by SIAs, by maternal Ab, not immune protected, and measles-susceptible cases in Chad from 2011 to 2025. The stacked bar chart depicted the immunity profile of preschool-aged children in Chad (Figure 2). According to the profile, the immunity gaps were 6%, 5%, and 6% in the cohort of children born in 2011, 2012, and 2013, respectively (Figure 2). In 2014 and 2015, 13% of the children born in the cohort were not immunized against measles (Figure 2). Our results showed an increase in population immunity gaps of 21% in 2016 and 2017 and 20% computed in 2018. In the same line, the 2016-2018 immunity gaps correlated with roughly four times more measles susceptibility cases than the first-year birth cohort. From 2019 to 2022, the immunity gaps ranged from 6% to 8%, correlating with a decrease of three to four times less than 2016-2018 (Figure 2). In 2023, 2024, and 2025, the population immunity gaps were estimated at 46%, 53%, and 38%, respectively, correlated to eight to 11 increased cases susceptible compared with 2016-2018 (Figure 2). Compared with the first-year cohort, children were approximately eight, nine, and six times less protected from measles in 2023, 2024, and 2025, respectively, increasing measles-susceptible cases by 12, 15, and 11 times (Figure 2). The overall cohort showed a significant cumulative cases of measles susceptibility. Our results also showed that children in each birth cohort were not protected against measles by vaccination because of suboptimal routine immunization coverage and the suboptimal coverage of the last SIA (Figure 2). The finding also showed inadequate MCV1 coverage, a delayed introduction of MCV2, low MCV2 coverage, and a high MCV1/MCV2 abandonment rate over time (Table 3).

**Table 2:** SIAs using measles-containing vaccines from 2012 to 2023 in Chad.

Year	Intervention	Start date	End date	Age group	Extent	Target population	Reached population	% Reached	Survey results	Areas/comments
2023	Measles	2023-12-05	2024-02-19	9-59 M	Sub-National	3 193 215	3 507 851	109.90	-	-
2022	Measles	2022-04-29	2022-05-05	9-59 M	Sub-National	73 129	74 596	102.00	-	-
2022	Measles	2022-04-05	2022-04-11	9-59 M	Sub-National	426 152	478 171	112.21	-	-
2021	Measles	2021-03-22	2021-03-28	9-59 M	Rollover-National	1 623 518	1 745 337	107.50	71.5	Bloc 2
2021	Measles	2021-01-12	2021-01-18	9-59 M	Rollover-National	1 792 830	1 950 968	108.80	82.8	Phase 1 of the national SIA with two phases in 66 districts in 10 provinces
2019	Measles	2019-12-12	2019-12-18	9 M-9 Y	Sub-national	424 728	467 455	110.10	-	-
2019	Measles	2019-06-26	2019-07-31	6 M-9 Y	Sub-national	113 409	103 543	91.30	-	-
2019	Measles	2019-06-01	2019-06-30	6 M-9 Y	Sub-national	102 144	95 198	93.20	-	-
2019	Measles	2019-04-09	2019-04-15	6 M-9 Y	Sub-national	618 739	653 535	105.60	-	-
2019	Measles	2019-01-01	2019-02-28	6 M-9 Y	Sub-national	99 882	106 965	107.10	-	-
2018	Measles	2018-11-17	2018-11-23	9 M-9 Y	Sub-national	2 415 484	2 421 067	100.00	90	Baher El Gazel, Borkou, Ennedi Est, Ennedi Ouest, Batha, Hadjer Lamis, Lac, Kanem, N’Djamena, Ouddai, and Wadi Fira
2017	Measles	2017-03-12	2017-03-18	9-59 M	Sub-national	693 526	707 103	102.00	82	Phase 2: nine regions
2016	Measles	2016-11-21	2016-11-27	9-59 M	Sub-national	2 083 306	2 342 341	112.40	82	Phase 1: 14 regions: Barh El Ghazal, Batha, Chari Baguirmi, Logone Occidental, Logone Oriental, Mandoul, Mayo kebbi Est, Mayo kebbi Ouest, Moyen Chari, N’Djamena, Tandjile, Kanem, Wadi Fira, Lac JRF 2017 reached: 2337633 Coverage survey report pending
2016	Measles	2016-05-25	2016-05-31	9 M-14 Y	Sub-national	418 517	414 392	99.00		conducted in seven districts
2014	Measles	2014-10-20	2014-10-26	6 M-9 Y	Sub-National	2 311 968	2 349 620	102.00	-	2nd phase - 38 districts
2014	Measles	2014-06-16	2014-06-22	6 M-9 Y	Sub-National	2 465 865	2 549 188	104.00	-	1st phase
2012	Measles	2012-01-16	2012-01-23	6-59 M	National	2 035 373	2 270 772	111.00	-	SIAs in two phases: 13 Regions 20-26 January. Nine Regions SIAs 27 January to 2 February 2012

M, month; SIAs, supplementary immunization activities; Y, year.

**Table 3:** Routine immunization coverage data were based on the World Health Organization and United Nations Children’s Fund estimates of National Immunization Coverage (WUENIC).

Year	MCV1(%)	MCV2(%)
2022	56	2
2021	55	0
2020	47	0
2019	41	0
2018	42	0
2017	37	0
2016	37	0
2015	46	0
2014	45	0
2013	57	0
2012	63	0
2011	54	0

MCV, measles-containing vaccine.

## Discussion

This study uses a Chadian birth cohort to depict the population measles immunization gaps in children aged 0-14 years from 2011 to 2025. We used the CDC methodology to model the percentage of immunity by MCV1, by MCV2, by maternal Abs, and measles by SIAs, as well as the proportion of measles-susceptible children categorized by year of cohort birth. Our findings revealed that children in each birth cohort were not protected against measles by vaccination because of suboptimal routine immunization coverage and the suboptimal coverage of the last SIA, increasing the measles susceptibility cases over time. The finding also showed inadequate MCV1 coverage, a delayed introduction of MCV2, low MCV2 coverage, and a high MCV1/MCV2 abandonment rate over time. A recent study, which supported our findings, found that measles vaccination coverage gaps are a frequent concern across Sahel countries, with MCV2 coverage lower than MCV1 [5]. Chad’s MCV2 coverage is extremely low, at only 35% in 2023; nevertheless, it has increased from 2% in 2022, when it was originally introduced [5]. Persistent measles immune gaps in all the birth cohorts from 2011 to 2025 could be the plausible explanation of recurring measles outbreaks because the measles-susceptible cases have exponentially increased in Chad. Studies have revealed that recurrent measles outbreaks were likely caused by an accumulation of unvaccinated, measles-susceptible children due to low MCV1 coverage and inefficient SIA implementation [8,26,27]. These disruptions to routine immunization services, mass campaigns, surveillance, and other core risk mitigation capacities have further exacerbated vulnerabilities to measles outbreaks worldwide [28]. As shown in Figure 2, the high population immunity gaps in children birth cohort 2016-2018 were corrected with a high number of measles-susceptible cases; the same observation was shown in the 2023-2025 birth cohort. A study found that future measles outbreaks could occur in local populations that may have more susceptible measles cases [3]. The persistent population immune gaps could be seen in the history of Chad, as shown in different studies [[29], [30], [31], [32]]. A recent modeling and forecasting of immunization against measles in Chad revealed that child immunization against measles is anticipated to vary between 39% and 59% per year over the next decade [33]. All these measles population immunity gaps, combined with the current humanitarian crisis caused by the influx of Sudanese refugees, concurrent epidemics, conflict, displacement, vaccine hesitancy, and other humanitarian crises that disrupt childhood vaccinations, making people more susceptible to measles, may worsen Chad’s recurrent measles outbreaks. This work calls on the Chadian government and partners to act quickly and systematically to identify sub-national population measles immunity gaps, allowing early planning of measles immunization operations to close those gaps.

Our findings may help explain the current situation of recurring measles outbreaks in other African nations with a low measles population immunity. According to recent data, measles vaccination coverage remained extremely low in 2019 in Angola (51%), Cameroon (60%), Central African Republic (49%), Chad (41%), Democratic Republic of Congo (57%), Ethiopia (58%), Guinea (47%), Somalia (46%), South Sudan (49%), Mali (59%), and Nigeria (54%) [5,34]. MCV2 was rarely part of routine schedules in countries eligible for support from the Global Alliance for Vaccines and Immunization until 2010 since introductions have accelerated with 60% WHO/UNICEF joint estimates of WUENIC MCV2 achieved by 2019 [35]. The level of measles population immunity required to sustain measles elimination (the so-called herd immunity threshold) is generally estimated to be above 90% [36]; hence, the WHO recommends that countries aiming at measles elimination should achieve ≥ 95% coverage with both doses equitably to all children in every district [35,37]. Each year, a substantial number of susceptible measles are identified in Chad and these susceptible individuals are responsible for the perpetuation of measles outbreaks by interacting with measles cases. For many years now, Chad has experienced several measles outbreaks, followed by poor response campaigns [38]. Routine vaccination programs in Chad have not been able to consistently provide a high proportion of children vaccinated against measles [30]. Moreover, vaccination programs in Chad make limited efforts to reach older children who failed to receive measles vaccine through routine services. The absence of recent SIAs, together with chronically low vaccine coverage, have combined to allow the numbers of children who are susceptible to measles to build up to very high levels and to precipitate these epidemics [30]. Furthermore, outbreak-response vaccination activities occurred very late in the epidemic in Chad [30,31]. Previous experience showed that it is important to note that SIAs targeting all children of between 9 months and 14 years have been implemented reaching 80% in Chad [30]. After, there were no measles epidemics reported, although we would not expect an epidemic immediately after a high-coverage SIAs or a major epidemic [30,31]. Although strengthening the routine immunization system to reach new cohorts sustainably, Chad should consider the implementation of appropriate catch-up vaccination strategies to reduce the probability of outbreaks in the future [4].The rapid fall in measles incidence in the Americas after the catch-up campaigns encouraged other regions to adopt these strategies, initially to pursue goals of measles mortality reduction and subsequently measles elimination [35]. A recent systematic review and meta-analysis of 14 studies conducted in low- and middle-income countries found that well-planned SIAs with genuine community engagement during the planning and implementation stages could potentially prevent a large number of measles cases, disabilities, and deaths [11]. Countries and stakeholders participating in childhood immunization campaigns can dramatically enhance measles epidemic planning and control by making evidence-based decisions [11]. One extremely useful strategy is to simulate the future outcomes and costs of alternative immunization schemes [11]. This meta-analysis found that vaccine-targeting interventions such vaccination reminders, cash incentives, community participation, and health education activities boost measles vaccination rates (relative ratio (RR) 1.19, 95% confidence interval 1.10-1.27) [11].

The strength of this study is that, to the best of our knowledge, it is the first of its genre to be conducted in Chad. The identification of population immunity gaps allows early planning of immunization activities to close immunity gaps. This study also has multiple limitations. In fact, the method for measuring measles immunity profile fails to account for geographical diversity within a country. Although immunity profiles can be generated for sub-national areas, such analyses are generally impossible because of a lack of coverage and population data at the subnational level, particularly, for SIAs. Because there are insufficient accurate coverage and population statistics at the sub-national level, the impact of each SIA is averaged at the national level, meaning that the country’s population will be totally mixed after each SIA. In practice, however, this mixing is rare, resulting in the considerable disparities in immunization program success observed at the sub-national level. Furthermore, the immunity profile does not account for immunity acquired by natural measles infection, which may explain why a large proportion of people with measles immunity in older age groups were born during a period when the disease was widespread. The quality of the input data, as well as the frequency and quality of nationally representative coverage surveys, all contribute to the analysis’ accuracy.

## Conclusion

Based on these findings, the measles population immunity gaps were found in all the birth cohorts from 2011 to 2025, increasing measles-susceptible cases over time. Our results also revealed that children in each birth cohort were not protected against measles by vaccination because of inadequate routine immunization coverage and suboptimal coverage of the last SIA. This finding could be explained by poor MCV1 coverage, a delayed launch of MCV2, low MCV2 coverage, and a high MCV1/MCV2 abandonment rate. Based on this, if the Chadian government and stakeholders do not implement concrete policies, the measles population immunity gaps may persist over time. The measles immunity gaps should be used for early planning of immunization activities to close immunity gaps in Chad. To prevent future measles outbreaks, MCV1 and MCV2 coverage must be improved to at least 95% at the national level and in all health districts each year, followed by the development of high-quality measles SIAs at the national and district levels. Once these data have been gathered, SIAs can be scheduled at extremely long intervals. To meet a new objective of eliminating measles in Chad by 2030, more efforts must be made to restore and increase surveillance efficacy and coverage using two MCV doses.

## Declarations of competing interest

The authors have no competing interests to declare.
